# Dataset with updated ozone depletion characterization factors for life cycle impact assessment

**DOI:** 10.1016/j.dib.2024.111103

**Published:** 2024-11-04

**Authors:** Anne E.M. van den Oever, Stefano Puricelli, Daniele Costa, Nils Thonemann, Maeva Lavigne Philippot, Maarten Messagie

**Affiliations:** a Electric Vehicle and Energy Research Group (EVERGi), Mobility, Logistics and Automotive Research Centre (MOBI), Department of Electric Engineering and Energy Technology (ETEC), Vrije Universiteit Brussel, Pleinlaan 2, 1050 Brussels, Belgium; b AWARE - Assessment on WAste and REsources, Department of Civil and Environmental Engineering, Politecnico di Milano, Piazza Leonardo da Vinci 32, 20133 Milano, Italy; c VITO/EnergyVille, Boeretang 200, 2400 Mol, Belgium; d Institute of Environmental Sciences (CML), Leiden University, 2300 RA Leiden, the Netherlands

**Keywords:** Ozone depletion potential (ODP), Brightway, Activity browser, SimaPro

## Abstract

This dataset provides the latest characterization factors for ozone depletion based on the most recent ozone depletion potentials from the 2022 World Meteorological Organization (WMO) scientific assessment. These characterization factors can be used in life cycle assessment (LCA) to convert emissions of ozone-depleting substances to the common unit of the ozone depletion impact category, measured in kg CFC-11-eq. The dataset is formatted for easy import into LCA software such as Brightway, the Activity Browser, and SimaPro. The characterization factors are provided for both 100-year and infinite time horizons. The dataset addresses the current limitations of life cycle impact assessment (LCIA) methods, which are outdated and lack comprehensive substance coverage, by including 318 substances reported in the latest WMO assessment. This update ensures relevance for current ozone depletion assessment, including substances banned but still in use, very short-lived substances, and N_2_O. The methodology for updating and converting characterization factors is provided, supporting future updates in line with new scientific assessments. The dataset aims to enhance the accuracy and comprehensiveness of ozone depletion impact assessments in LCA studies.

Specifications Table

**Table:** 

Subject	Environmental engineering
Specific subject area	Characterization factors for ozone depletion in life cycle impact assessment
Type of data	Table (.xlsx format and .csv format) Supporting materials (Jupyter notebooks for import in Brightway and the Activity Browser software)
Data collection	Characterization factors with an infinite time horizon were directly obtained from ozone depletion potentials (ODPs) reported in the literature. The steady-state ODPs derived from the literature were converted to time-dependent characterization factors with a 100-year time horizon.
Data source location	Institution: Vrije Universiteit Brussels (VUB) Primary data sources: WMO 2022 scientific assessment of ozone depletion; ReCiPe 2016
Data accessibility	Repository name: Zenodo Data identification number: 10.5281/zenodo.12818532 Direct URL to data: https://doi.org/10.5281/zenodo.12818532
Related research article	A.E.M. van den Oever, S. Puricelli, D. Costa, N. Thonemann, M. Lavigne Philippot, M. Messagie, Revisiting the challenges of ozone depletion in life cycle assessment, Cleaner Environmental Systems 13 (2024) 100,196. 10.1016/j.cesys.2024.100196.

## 1 Value of the Data

• The dataset contains characterization factors for ozone depletion based on 318 most updated ozone depletion potentials (ODPs) published in the latest scientific assessment report on ozone depletion.
• The dataset is provided in various formats to allow direct import in widely-used life cycle assessment (LCA) software: Brightway, the Activity Browser, SimaPro, and potentially in other software like openLCA.
• Characterization factors are provided for both a 100-year and infinite time horizon, allowing for different time perspectives in life cycle impact assessment.
• The method is replicable, allowing for future updates following the scientific assessment reports on ozone depletion.

## 2 Background

In a previous paper [1], the challenges of ozone depletion in life cycle assessment were reviewed, highlighting that current life cycle impact assessment (LCIA) methods are outdated and lack substance coverage [1]. The most updated methods, IMPACT World+ [2] and Environmental Footprint (EF) 3.1 [3,4], use ozone depletion potential (ODP) values from the World Meteorological Organization (WMO) scientific assessment of ozone depletion [5] from 2014. However, since then, two new WMO assessments have been published. Moreover, the IMPACT World+ and EF 3.1 methods cover only 25 and 23 substances, respectively, while the latest WMO assessment [6] reports 318 substances. Although 93 of these substances are banned by the Montreal Protocol (MP), emissions could still occur due to exempt applications and recycling in existing equipment [7]. Other substances not covered by IMPACT World+ and EF 3.1 also remain relevant for assessing ozone depletion. For example, very short-lived substances, such as CH_2_Cl_2_ and CHCl_3_, are growing in atmospheric abundance, and their release is expected to rise due to the growing need for solvents and chemical feedstocks [1]. Finally, IMPACT World+ and EF 3.1, along with most other impact assessment methods, neglect N_2_O, the main anthropogenic contributor to ozone depletion today [8].

## 3 Data Description

**Fig. 1:**
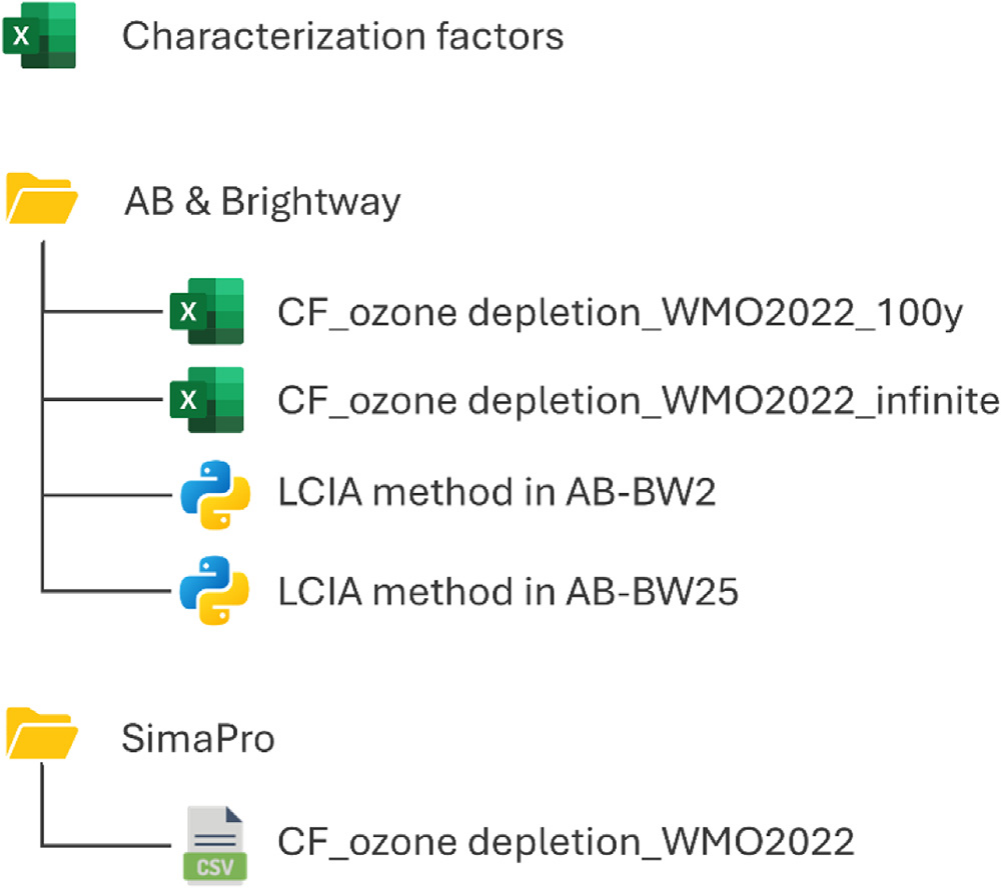
Overview of repository structure.

Considering the limitations of currently available LCIA methods for ozone depletion, this dataset presents an updated method for ozone depletion, using the 318 ODPs reported in the 2022 WMO scientific assessment of ozone depletion as midpoint characterization factors. The method consists of two impact categories, one considering an infinite time horizon, as originally reported by WMO, and one with a 100-year time horizon. The characterization factors are provided in the accompanying Excel file “Characterization factors” (Fig. 1). In addition, the file provides the controlled status by the MP [7] and the characterization factors used by the most common LCIA methods [1] for comparison.

To import the method in the Activity Browser or Brightway, a spreadsheet for each time perspective is available in the “AB & Brightway” folder. In addition, two python files are available, compatible with brightway2 and brightway2.5, to facilitate the import. The files are designed to be user-friendly, requiring no programming skills from the user. The code first identifies the substances that are not present in the biosphere database. These substances are then added to the biosphere database, after which the characterization factors are imported as a new method.

In the “SimaPro” folder, a CSV file is available to import the method in SimaPro, containing the method from both time perspectives. The substance names in the CSV file follow the SimaPro naming convention in case a substance was already present in SimaPro; otherwise, a new substance was created.

## 4 Experimental Design, Materials and Methods

The characterization factors of the LCIA methods reviewed in [1] were directly obtained from the most recent documentation available for each method.

The characterization factors for the updated method with an infinite time horizon were directly obtained from the steady-state ODPs from the 2022 WMO assessment. When an upper value was reported (e.g., *ODP_inf,__x_* < 0.02), this upper value was used as a characterization factor. When a range was reported, the average value was taken. To convert the steady-state ODPs from the 2022 WMO assessment to time-dependent ODPs, Eqs. (1) and (2) were used, following [9]:

(1) $$ODP_{t,x}=\;ODP_{inf,x}*\frac{F_{t,x}}{F_{t,\;CFC-11}}$$

(2) $$F_{t}=1-e^{\left(-t-3\right)*k}$$

Where *ODP_t,x_* is the ODP of substance *x* with a time horizon of *t (=100)* years, *ODP_inf,x_* is the steady-state ODP of substance *x* as reported by WMO [6], *F_t_* is the fraction of the total damage caused by substance *x* during the first *t* years, and *k* is the removal rate of substance *x* in y^-1^. The removal rate *k* is the inverse of the atmospheric lifetime retrieved for each substance from WMO [6].

In addition to the 318 WMO ODPs, the ODP for a generic mix of unspecified ODSs, “hydrocarbons, chlorinated” was derived from the Recipe 2016 method [10], as this flow is used in various datasets in the ecoinvent database [11]. The hierarchal perspective was used for the 100-year time horizon, while the egalitarian perspective corresponded to the infinite time. Thus, in total, the dataset contains 319 characterization factors.

As all files are human-readable, the methodology is easily replicable by updating the values manually, allowing for regular updates following the four-yearly scientific assessment reports published by the WMO. For the Activity Browser and Brightway, the import notebook is designed to allow new substances to be directly added to the spreadsheets. Through the appropriate commands in the software, new substances or elementary flows can be added, into SimaPro and then added to the LCIA method together with their value. The import in Activity Browser 2.9.4, Brightway2 2.4.4, Brightway25 1.0.6, and SimaPro 9.5.0.2 was successfully tested.

## Limitations

The proposed LCIA methods might not be fully compatible with future releases of the addressed software. You are encouraged to contact the corresponding author to report any compatibility issues.

## Ethics Statement

All authors have read and followed the ethical requirements for publication in Data in Brief and confirm that the current work does not involve human subjects, animal experiments, or any data collected from social media platforms.

## CRediT Author Statement

**Anne van den Oever:** Conceptualization, Methodology, Data curation, Formal analysis, Validation, Writing – Original draft, **Stefano Puricelli:** Conceptualization, Methodology, Data curation, Formal analysis, Visualization, Writing – Original draft, **Daniele Costa:** Validation, Supervision, Writing – Review & Editing, **Nils Thonemann:** Formal analysis, Validation, Writing – Review & Editing, **Maeva Lavigne Philippot:** Writing – Review & Editing, **Maarten Messagie:** Supervision, Funding acquisition.

## Data Availability

[12]Life Cycle Impact Assessment method for ozone depletion based on WMO 2022.
